# Correlation between local instantaneous dose rate and oxygen pressure reduction during proton pencil beam scanning irradiation

**DOI:** 10.1016/j.phro.2024.100614

**Published:** 2024-07-20

**Authors:** Eleni Kanouta, Jacob Graversen Johansen, Sara Poulsen, Line Kristensen, Brita Singers Sørensen, Cai Grau, Morten Busk, Per Rugaard Poulsen

**Affiliations:** a Danish Centre for Particle Therapy, Aarhus University Hospital, Aarhus, Denmark; b Department of Clinical Medicine, Faculty of Health, Aarhus University, Aarhus, Denmark; c Department of Physics and Astronomy, Aarhus University, Aarhus, Denmark; d Department of Experimental Clinical Oncology, Aarhus University Hospital, Aarhus, Denmark; e Department of Oncology, Aarhus University Hospital, Aarhus, Denmark

**Keywords:** Oxygen dynamics, Phosphorescence quenching, Proton pencil beam scanning, Oxygen consumption g-value

## Abstract

**Background and purpose:**

Oxygen dynamics may be important for the tissue-sparing effect observed at ultra-high dose rates (FLASH sparing effect). This study investigated the correlation between local instantaneous dose rate and radiation-induced oxygen pressure reduction during proton pencil beam scanning (PBS) irradiations of a sample and quantified the oxygen consumption g-value.

**Materials and methods:**

A 0.2 ml phosphorescent sample (1 μM PtG4 Oxyphor probe in saline) was irradiated with a 244 MeV proton PBS beam. Four irradiations were performed with variations of a PBS spot pattern with 5 × 7 spots. During irradiation, the partial oxygen pressure (pO_2_) was measured with 4.5 Hz temporal resolution with a phosphorometer (Oxyled) that optically excited the probe and recorded the subsequently emitted light. A calibration was performed to calculate the pO_2_ level from the measured phosphorescence lifetime. A fiber-coupled scintillator simultaneously measured the instantaneous dose rate in the sample with 50 kHz sampling rate. The oxygen consumption g-value was determined on a spot-by-spot level and using the total pO_2_ change for full spot pattern irradiation.

**Results:**

A high correlation was found between the local instantaneous dose rate and pO_2_ reduction rate, with a correlation coefficient of 0.96–0.99. The g-vales were 0.18 ± 0.01 mmHg/Gy on a spot-by-spot level and 0.17 ± 0.01 mmHg/Gy for full spot pattern irradiation.

**Conclusions:**

The pO_2_ reduction rate was directly related to the local instantaneous dose rate per delivered spot in PBS deliveries. The methodology presented here can be applied to irradiation at ultra-high dose rates with modifications in the experimental setup.

## 1 Introduction

Tissue oxygenation plays an important role in radiotherapy, where hypoxia is known to induce radio resistance in tissue [1]. Radiation can impact the tissue oxygen level, but this is normally not of concern in conventional dose rate radiotherapy, where the relatively slow radiation-induced oxygen consumption in tissue is compensated by re-oxygenation. The interest in radiation-induced oxygen-changes has been re-ignited recently due to the preferential normal tissue sparing (FLASH sparing effect) of radiation with ultra-high dose rates [2].

The role of oxygen in the induction of the FLASH effect has been strongly debated, and the underlying mechanism is not fully understood [3]. While oxygen depletion models may explain toxicity variations observed across a wide range of beam parameters in animal experiments [4], the actual oxygen consumption observed during FLASH irradiation seems too small to induce radioprotection [5]. While the FLASH effect cannot be directly explained by the oxygen consumption to radioprotective levels, the oxygen level might still play a role. An investigation of the radiation-induced oxygen consumption could therefore provide useful insights on the FLASH effect. A non-invasive method to investigate the oxygen dynamics under different irradiation scenarios is phosphorescence quenching, which has been used for both electron beams [6], [7] and passive scattered proton beams [8], [9], [10] with different dose rates. This method entails measurement of the phosphorescence decay from a molecular probe whose phosphorescence quenching is highly selective for oxygen. The soluble molecular probe can be used for partial oxygen pressure (pO_2_) measurements in sample solutions or injected directly into tissue. The pO_2_ measurements can be used to derive the oxygen consumption per unit of delivered dose, known as the g-value, under different experimental conditions.

In this study, we used phosphorescent probing to measure the pO_2_ dynamics in real-time in a phosphorescent probe solution during irradiation of a sample with proton pencil beam scanning (PBS). The correlation between simultaneous measurements of the local dose rate and pO_2_ changes was investigated and used to derive the oxygen consumption g-value for proton PBS.

## 2 Materials and methods

The study consisted of a pO_2_ calibration of a molecular probe followed by four proton PBS irradiations of a sample with the probe with simultaneous measurements of the dose rate and the pO_2_ level. Afterwards, the oxygen consumption g-value was determined by relating the measured dose rates with the pO_2_ reduction rate.

### 2.1 pO_2_ calibration

A calibration to relate the measured phosphorescence decay time of the PtG4 molecular probe [11] to the pO_2_ level was performed, as the calibration provided from the manufacturer (for pH=7.2 and T=22.5 °C) resulted in an unrealistically high pO_2_ level of 180 mmHg, when the sample was in equilibrium with the atmosphere. The expected pO_2_ level under atmospheric conditions, based on the temperature and pressure of the given day, was approximately 155 mmHg.

The phosphorescence lifetime of the sample containing the molecular probe was measured using a phosphorometer (Oxyled, Oxygen Enterprises, Ltd.). The details regarding the phosphorometer have been described previously [6], [8], [9] while a brief overview of the system’s operation is presented next.

For the calibration, 5 ml of a sample with 1 µM of PtG4 molecular probe in saline (isotonic solution, 9 mg/ml NaCl, pH=6, T=23.5 °C) was placed in a glass tube. A gas of known oxygen concentration was bubbled through the sample, while the phosphorescence lifetime was measured continuously by the phosphorometer. This was done by repeatedly exciting the sample by 10 µs light pulses from a red LED after which the emitted phosphorescent light from the probe was measured for 480 µs. The excitation was repeated every 500 µs. The LED light was directed to the sample using an optical fiber while the phosphorescence light was directed through a second optical fiber to a photodetector (avalanche photodiode).

The calibration was performed by bubbling gasses consisting of 0 %, 0.5 %, 2 %, 5 % or 19.5 % O_2_, with 5 % CO_2_ and 75.5–95 % N_2_ through the sample. The gas was bubbled through the sample, while the phosphorescence lifetime was measured by the phosphorometer continuously. Once, a stable phosphorescence lifetime was reached (constant lifetime over 60 s), the bubbling was stopped and a phosphorescence lifetime was measured without bubbling. It was assumed that equilibrium in the O_2_ level of the gas and the sample was established at this point, and that the O_2_-level was homogenous across the entire sample, owing to the constant bubbling during outgassing, hence eliminating volume effects.

The mean phosphorescence lifetime in the equilibrium was calculated based on a single-exponential fit to the sum of 200,000 decay curves. The O_2_ concentration of the sample was converted to pO_2_ based on the environmental conditions (temperature, pressure) at the time of the experiment. Calibration parameters for translation of the phosphorescence lifetime (τ) to pO_2_ were determined according to the following equation (Stern-Volmer model) [11]:

(1) pO2=1τ-1τ0kq

where τ_0_ is the phosphorescence lifetime in the absence of oxygen and k_q_ is the quenching constant of the phosphorescence state. τ_0_ and k_q_ were determined by a linear fit of pO_2_ as a function of 1/τ.

### 2.2 PBS irradiation

**Fig. 1:**
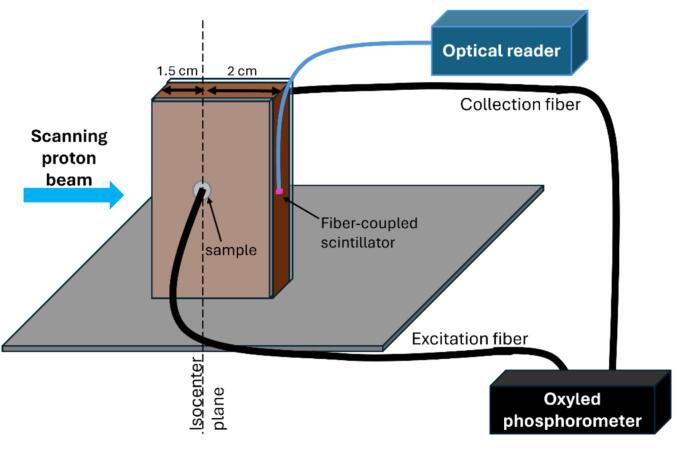
Side view of the experimental setup for simultaneous pO_2_ and instantaneous dose rate measurements. During proton PBS scanning LED light pulses from the Oxyled phosphorometer excited the molecular probe in the sample solution through an optical fiber (excitation fiber). The subsequent phosphorescent decay light was sent to the phosphorometer through another fiber (collection fiber). A fiber-coupled scintillator measured the instantaneous dose rate in a point on the exit side of the phantom.

Simultaneous dynamic pO_2_ and dose rate measurements were performed in real time during PBS irradiation of a 0.2 ml saline sample solution (isotonic solution, 9 mg/ml NaCl) containing Oxyphor PtG4 (1 μM) (Fig. 1). The calibration parameters from Section 2.1 were used. The sample was placed in a sealed cylindrical compartment (5 mm diameter, 10 mm height) made by drilling a 5 mm hole in a 10 mm thick solid water block. The compartment was then sealed on each side by 3 mm thick PMMA plates (Fig. 1). The irradiation took place at the fixed horizontal proton beamline (ProBeam, Varian, a Siemens Healthineers company, Palo Alto, CA, USA) at the Danish Centre for Particle Therapy, Aarhus University Hospital, Denmark.

**Fig. 2:**
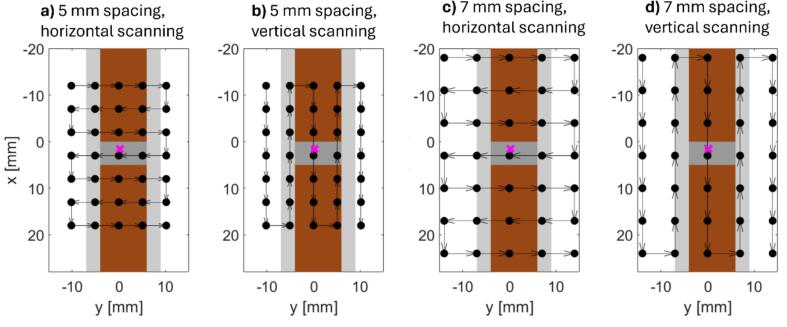
The four applied PBS fields with different spot spacing and scanning direction. The PBS fields are shown in the isocenter plane in beam’s eye view. The position of the solid water phantom (brown), PMMA plates (light grey) and sample volume (dark grey) as well as the position of the fiber-coupled scintillator (magenta) relative to the PBS field are indicated in each case. (For interpretation of the references to colour in this figure legend, the reader is referred to the web version of this article.)

Four irradiations with varying PBS fields were performed. The fields consisted of 5 × 7 spots with either 5 mm or 7 mm spacing using both horizontal or vertical scanning directions (Fig. 2). The spot pattern resembled the one previously used in pre-clinical murine proton FLASH studies at our institution [12], [13]. The irradiation was delivered with a 244 MeV transmission proton beam with the center of the sample aligned to the isocenter at a depth of 1.5 cm (Fig. 1). The field dose rate was 1.2–2.7 Gy/s and the maximum instantaneous dose rate was approximately 40 Gy/s. High field doses of 64–120 Gy were used in order to induce substantial pO_2_ changes in the sample. Table 1 presents information about each irradiation.

**Table 1:** Dose and field dose rate for each PBS field and resulting g-values. The g-values were determined on a spot-by-spot level for each irradiation from the linear fit of the data in Fig. 5 and reported as the fitted slope (with 95 % confidence interval). The g-values for the total irradiations are also reported for each PBS field. The resulting mean value (±standard deviation) from all measurements for the two calculation methods is shown in the last row.

**PBS field**	**Dose to sample volume** **(Gy)**	**Field dose rate (Gy/s)**	**g-value (mmHg/Gy)**	
			**Spot-by-spot**	**Total irradiation**
5 mm spacing, horizontal scanning (Fig. 2a)	118	2.7	0.18 (0.15, 0.20)	0.17
5 mm spacing, vertical scanning (Fig. 2b)	120	2.1	0.19 (0.18, 0.21)	0.17
7 mm spacing, horizontal scanning (Fig. 2c)	70	1.3	0.19 (0.17, 0.20)	0.16
7 mm spacing, vertical scanning, (Fig. 2d)	64	1.2	0.17 (0.14, 0.20)	0.18
All measurements			0.18 ± 0.01	0.17 ± 0.01

### 2.3 pO_2_ measurements

**Fig. 3:**
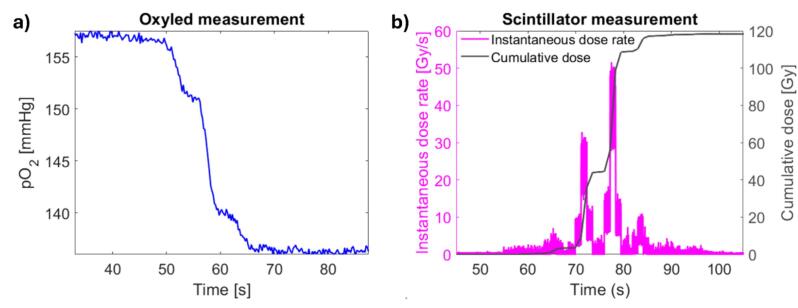
The (a) partial oxygen pressure (pO_2_) and (b) instantaneous dose rate and cumulative dose in the scintillator position measured as a function of time for the PBS field in Fig. 2a.

The partial oxygen pressure in the sample was measured with the phosphorometer during the irradiations. The two fibers from the phosphorometer directly touched the PMMA plates on each side of the sample (Fig. 1). The sample was excited by 5 µs light pulses after which the phosphorescent light from the probe was measured for 235 µs. The excitation was repeated every 250 µs and for each sampling, the decay curves of 500 pulses were summed and used to determine the phosphorescence lifetime by a single-exponential fit. Due to the small sample volume and the placement of the fibers (4 mm diameter at 3 mm distance from the sample volume) it was assumed that the whole sample volume was illuminated homogeneously. Using Eq. (1) this resulted in pO_2_ being measured with a sampling rate of 4.5 Hz. It resulted in 4–9 samplings per spot as the spot duration ranged from 1.1 s to 2.1 s. An example of the measured pO_2_ for the PBS field of Fig. 2a is shown in Fig. 3a.

### 2.4 Dosimetry

Simultaneously with the pO_2_ measurements, the instantaneous dose rate in a point 2 cm behind the sample was measured with an in-house developed fiber-coupled scintillator detector [14]. The fiber-coupled scintillating crystal (ZnSe:O crystal with sub-millimeter dimensions (ISMA, Ukraine)) was placed on the exit side of the solid water phantom, approximately at the center of the sample (Fig. 1). The light emitted by the scintillator upon irradiation was guided through an optical fiber to a Si photomultiplier (MicroFC-SMTPA-60035, SensL), resulting in the instantaneous dose rate sampled at a rate of 50 kHz (see example in Fig. 3b). The detector was previously calibrated for absolute instantaneous dose rate measurements in the entrance plateau of monoenergetic high energy proton beams [15]. Using machine log files containing the timing of each proton spot, each instantaneous dose rate measurement was assigned to individual proton spots.

### 2.5 Correlation between pO_2_ and local instantaneous dose rate

To relate the measured pO_2_ changes to the total accumulated dose in the sample, the point dose rate measurement at the position of the scintillator detector (Fig. 3b) was used to estimate the cumulative dose in the sample volume. The temporal resolution of the detector allowed for measurement of the dose rate per spot at the detector position (mean signal in each signal plateau in Fig. 3b). The detector position was then determined through a fit of the dose rates as function of spot position to a two-dimensional Gaussian function. The detector position being the top-point of the Gaussian function, and the Gaussian width being the beam spot width [15]. The fit was performed using the planned spot positions, as previous measurements using scintillation imaging have shown good agreement between planned and delivered spot positions [16].

Next, the dose delivered in the sample volume by each spot in the PBS field was calculated by integrating the assumed Gaussian spot dose profile over the sample volume. Here, the spot width in the sample depth was assumed to be equal to the width measured at the scintillator detector depth. Finally, the spot doses were combined with spot durations in machine log files [14] to determine the cumulative mean dose in the sample as function of time.

The resulting time-resolved cumulative dose was synchronized to the pO_2_ measurements by aligning the time of 50 % pO_2_ reduction with the time of 50 % dose delivery. This directly provided the rate of pO_2_ change (dpO_2_/dt) and the mean dose rate for each spot.

The oxygen consumption g-value, i.e. the pO_2_ reduction per dose, was determined in two different ways. First, dpO_2_/dt as a function of the dose rate per spot were fitted to a linear function and the slope of the fit gave the g-value for each irradiation. Due to low signal-to-noise ratio, spots with dose rate less than 0.5 Gy/s were excluded. Next, the g-value was also calculated using the total reduction in pO_2_ and the total delivered dose for the entire field delivery, for each irradiation. Furthermore, the impact of the initial pO_2_ level on the measured g-value was investigated.

## 3 Results

### 3.1 pO_2_ calibration

The calibration resulted in fitting parameters in Eq. (1) of τ_0_ = 48.7 ± 0.7 µs and k_q_ = 233.6 ± 7.2 mmHg^−1^ s^−1^. For comparison, the parameters of the calibration provided by the manufacturer were: τ_0_ = 52.02 µs and k_q_ = 194.4 mmHg^−1^ s^−1^.

### 3.2 Correlation between pO_2_ and local instantaneous dose rate

**Fig. 4:**
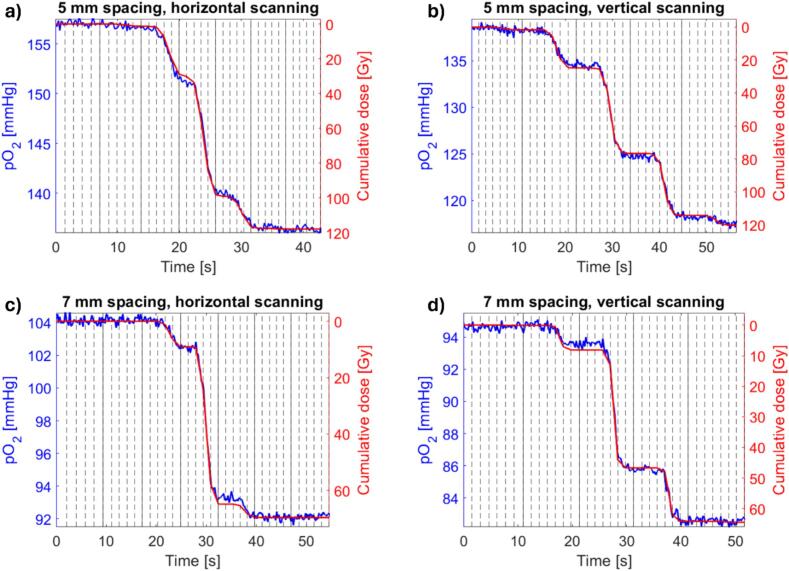
Measured pO_2_ (left axis) and reconstructed mean cumulative dose in the sample (right axis) after synchronization for the four PBS fields shown in Fig. 2. The axis for the cumulative dose is inverted to highlight the correlation between the two curves. The vertical lines mark the transition between beam spots with solid lines indicating the change between rows for horizontal scanning and between columns for vertical scanning.

**Fig. 5:**
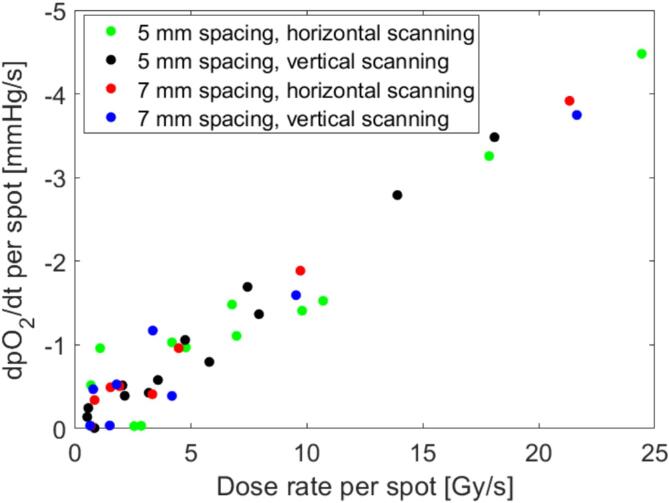
Rate of change in partial oxygen pressure pO_2_ (dpO_2_/dt) versus mean dose rate for each spot for all four PBS fields. The correlation coefficient was 0.96–0.99.

The pO_2_ change, which synchronized well with the cumulative mean dose in the sample, is shown for all four PBS fields in Fig. 4. The dpO_2_/dt and dose rate per spot for all four PBS fields showed a high linear correlation (Fig. 5), with a correlation coefficient of 0.96–0.99.

The g-values were 0.18 ± 0.01 mmHg/Gy (mean ± standard deviation) when calculated on a spot-by-spot level from linear fits of the data in Fig. 5 and 0.17 ± 0.01 mmHg/Gy when calculated from the total irradiation (Table 1). The initial pO_2_ level before each PBS field delivery was 156–94 mmHg. No trend was found for the g-value as a function of the initial pO_2_ level.

## 4 Discussion

In this study, we presented a methodology for simultaneously measuring O_2_ dynamics and dose rate on a spot-by-spot time scale during PBS irradiation of a small sample. The rate of pO_2_ reduction was highly correlated with the local instantaneous dose rate delivered to the sample by the scanning pencil beam and was used to quantify the oxygen consumption g-value.

Measurement of the pO_2_ level using phosphorescence quenching have been previously used for real-time measurements in electron [6], [7] and scattered proton beams [8], [9], [10], showing a linear drop in the pO_2_ level during the beam delivery. Furthermore, the total radiation-induced pO_2_ reduction caused by proton PBS was reported recently, however without any details on the time structure of the pO_2_ changes or its relationship to the delivery of individual spots [17]. As demonstrated in the current study, the complex time structure of the local dose deposition in a volume by the narrow scanning beam causes highly correlated changes in the local pO_2_ level in the volume (Fig. 4).

The oxygen consumption g-value was found here either on a spot-by-spot level or for a field delivery. This yielded very similar g-values of 0.18 ± 0.01 mmHg/Gy and 0.17 ± 0.01 mmHg/Gy, respectively (Table 1). The g-values did not show any trend as a function of the initial pO_2_ level of the sample, which is in agreement with previous studies [8], [9] for the range of the pO_2_ levels used (156–94 mmHg). These previous studies showed a decrease in the g-value when the pO_2_-level went below 20 mmHg. A confirmation of this was beyond the scope of the current study. The g-values are in agreement with previously reported values for measurements in aqueous solutions, which ranged from 0.19 to 0.55 mmHg/Gy for conventional dose rates (0.1–0.6 Gy/s field dose rate) [6], [8], [9] depending on the solution and the parameters of the beam delivery. The g-values, in the same studies, were found to be lower for ultra-high dose rates (100–300 Gy/s field dose rate) than for conventional dose rates. In this study, only conventional dose rates were used, but no significant change was observed for the g-value in the range of 0 to 25 Gy/s dose rates (Fig. 5). It would be interesting to use the methodology for ultra-high dose rate proton PBS where local instantaneous dose rates >1000 Gy/s are readily obtainable [15]. This could be used for detailed mapping of the g-value as function of dose rate, which would be a valuable addition to previous studies that compared only one conventional dose rate and one ultra-high dose rate [6], [7], [8], [9], [17].

The pO_2_ values measured during irradiation ranged from 156 mmHg to 83 mmHg when the calibration performed as part of this study was used. The pO_2_ levels based on the manufacturer calibration were 12–13 % higher than the values reported here. As the calibration depends on the pH level of the sample and the temperature [11], it could potentially be the reason from the deviation between our calibration and the manufacturer calibration. If we had used the manufacturer calibration the reported g-values would have been 10–15 % higher than reported in Table 1.

The current study was limited to PBS deliveries with high doses (64–120 Gy), which were needed to induce considerable changes per spot in the pO_2_ level due to the low g-values (Table 1). Moreover, relatively low dose rates (field dose rate of 1.2–2.7 Gy/s) were used in the current study, while the methodology can be extended to higher dose rates. Since the applied fiber-coupled scintillator has 50 kHz resolution and has been calibrated for instantaneous dose rates of >1000 Gy/s [15], it can readily be used for higher instantaneous dose rates without any changes. Furthermore, the sampling time of the phosphorometer can be increased considerably at the cost of lower signal-to-noise levels by shortening the time interval used for collecting the phosphorescence light and by averaging over fewer excitation pulses than the 250–500 pulses used in this study. This has been previously been used to obtain sampling rates for up to 3.33 kHz [8].

Regardless of the limitations of the current study, the methodology described here could be useful in quantifying the pO_2_ changes from different irradiation scenarios such as different doses, dose rates and different temporal beam deliveries [12], [13], as well as during murine studies, where the combined instantaneous oxygenation and re-oxygenation can be measured to investigate if an effect can be seen. However, instantaneous dose rates significantly higher than 25 Gy/s are required, as evident from this study.

In conclusion, a methodology for simultaneous measurements of pO_2_ and dose rate was presented that allowed quantification of the oxygen consumption g-values either on a field or on a spot level for proton PBS. In the PBS irradiation, the pO_2_ reduction rate in a small sample was directly related to the local instantaneous dose rate in the sample for a range of PBS fields.

## Declaration of Competing Interest

The authors declare that they have no known competing financial interests or personal relationships that could have appeared to influence the work reported in this paper.
